# Evidence that the Malaria Parasite *Plasmodium falciparum* Putative Rhoptry Protein 2 Localizes to the Golgi Apparatus throughout the Erythrocytic Cycle

**DOI:** 10.1371/journal.pone.0138626

**Published:** 2015-09-16

**Authors:** Stéphanie Hallée, Dave Richard

**Affiliations:** Centre de recherche en infectiologie, CHU-Université Laval, Quebec City, Quebec, Canada; Ehime University, JAPAN

## Abstract

Invasion of a red blood cell by *Plasmodium falciparum* merozoites is an essential step in the malaria lifecycle. Several of the proteins involved in this process are stored in the apical complex of the merozoite, a structure containing secretory organelles that are released at specific times during invasion. The molecular players involved in erythrocyte invasion thus represent potential key targets for both therapeutic and vaccine-based strategies to block parasite development. In our quest to identify and characterize new effectors of invasion, we investigated the *P*. *falciparum* homologue of a *P*. *berghei* protein putatively localized to the rhoptries, the Putative rhoptry protein 2 (PbPRP2). We show that in *P*. *falciparum*, the protein colocalizes extensively with the Golgi apparatus across the asexual erythrocytic cycle. Furthermore, imaging of merozoites caught at different times during invasion show that PfPRP2 is not secreted during the process instead staying associated with the Golgi apparatus. Our evidence therefore suggests that PfPRP2 is a Golgi protein and that it is likely not a direct effector in the process of merozoite invasion.

## Introduction

Invasion of an erythrocyte by the extracellular malaria merozoite is an essential multistep process mediated by a set of molecules distributed on the parasite surface and within specialized apical organelles (the rhoptries, micronemes and dense granules). These organelles are secreted in a coordinated, stepwise manner according to their respective roles in the invasion process. Micronemal proteins are involved in attachment to the red blood cell, tight junction (TJ) formation and as adhesins through which the merozoite propels itself through the action of an actomyosin motor [1–3]. Rhoptry proteins are involved in initial recognition and TJ formation but also in the generation of the parasitophorous vacuole and the modification of the host cell [4]. Finally, dense granule proteins are involved in host cell modification [5–7]. Interestingly, this compartmentalization is also observed in the rhoptry organelle itself with proteins in the rhoptry tip, neck, bulb and membrane being sequentially secreted [8].

More than 30 rhoptry proteins have been identified in *P*. *falciparum* to date [9]. Proteomic analysis of isolated rhoptries from rodent malaria parasites identified 36 potential rhoptry proteins, 18 of which were novel and possessed orthologues in *P*. *falciparum* [10, 11]. Whether these truly localize to the *P*. *falciparum* rhoptries remains to be investigated. The *P*. *berghei* Putative rhoptry protein 2 (PbPrp2, PlasmoDB: PBANKA_141830) is a homologue of a putative *Toxoplasma gondii* rhoptry protein identified through a proteomic analysis of isolated rhoptries [12] that was shown to localize to the apical tip of merozoite forms [13]. PbPrp2 was also found in ring and trophozoite stage parasites, potentially at the Golgi apparatus, a pattern reminiscent of other rhoptry proteins expressed before the appearance of these organelles [14–16]. Intriguingly, the protein did not localize to the rhoptries of mature salivary gland sporozoites instead being found at the ER/Golgi[13]. The inability to inactivate the *Pbprp2* gene suggested that it plays a critical role in the erythrocytic cycle[13]. In our efforts to study the invasion process of *P*. *falciparum* merozoites, we decided to investigate the *P*. *falciparum* homologue of PbPRP2. We here present evidence that PfPRP2 extensively colocalizes with the Golgi apparatus throughout the erythrocytic cycle and not with the rhoptries. Furthermore, we show that PfPRP2 is not secreted during the invasion process contrarily to known rhoptry proteins.

## Results and Discussion

### PfPRP2 is a *Plasmodium* specific protein

**Fig 1:**
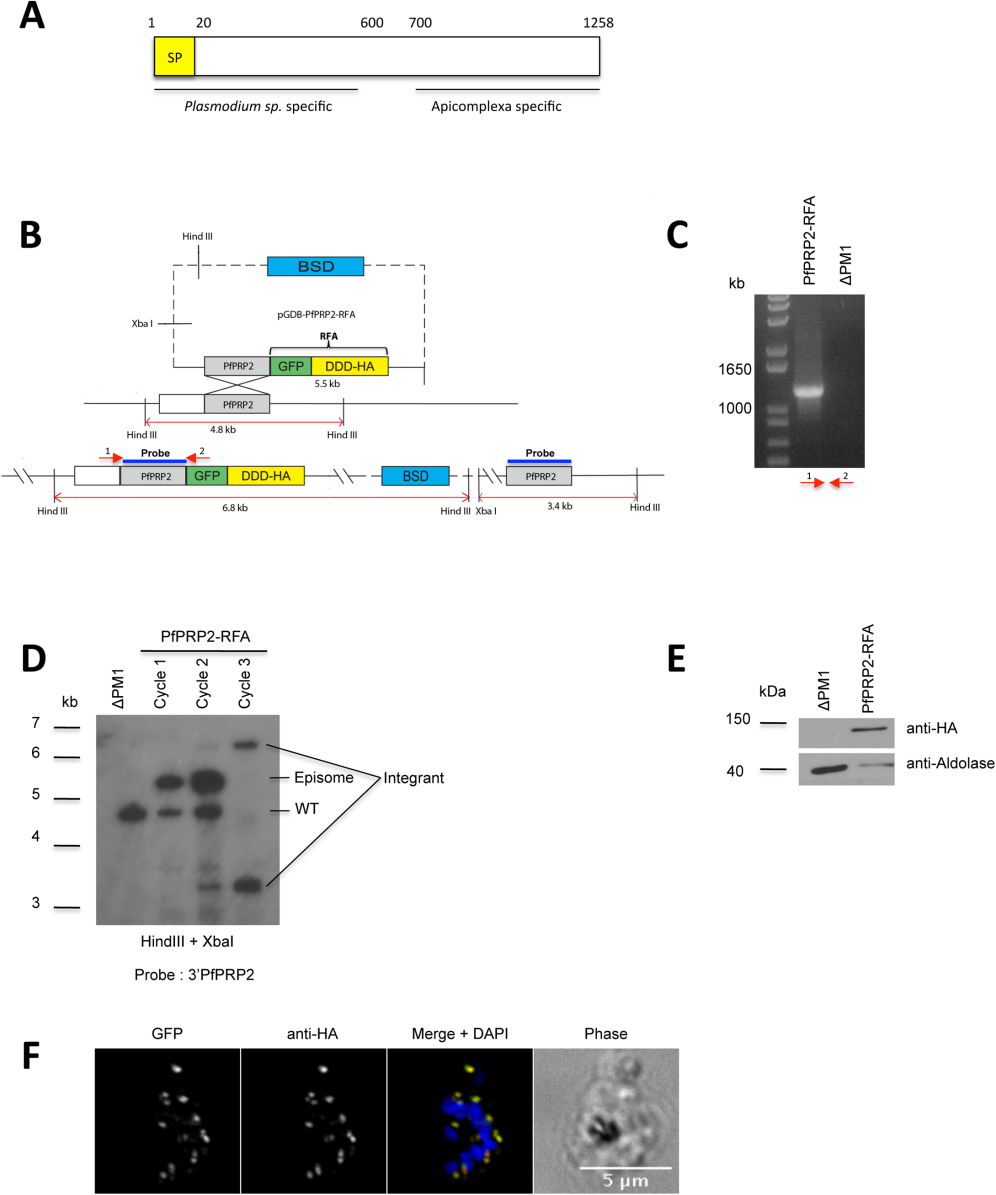
Generation of a PfPRP2-RFA tagged parasite line. (A) Domain organization of PfPRP2. BLAST analysis reveals that the N-terminus is only present in *Plasmodium* species whilst the C-terminus is conserved in a number of apicomplexans. The conserved regions were identified using the full length PfPRP2 sequence through protein BLAST at http://blast.ncbi.nlm.nih.gov/Blast.cgi. SP: Signal peptide (B) Schematic of the strategy used to integrate the RFA system at the 3’end of PfPRP2 by single crossover homologous recombination. The expected sizes of digested products for Southern blot are shown. *DDD*, DHFR degradation domain; *BSD*, Blasticidin. C) PCR confirming integration of the RFA system at the PfPRP2 endogenous locus. Primers used for amplification are indicated by red arrows. (D) Southern blot showing integration of the RFA cassette at the 3’ of the PfPRP2 locus and the loss of the episomal form of pGDB-PfPRP2-RFA and the wild type PfPRP2 gene after three BSD selection cycles. The 3'PfPRP2 probe consists of nucleotides 2819 to 3774 of PfPRP2. (E) Western blot using a mouse monoclonal anti-HA confirms the expression of PfPRP2-RFA in the tagged line. Anti-Aldolase is used as loading control. (F) IFA using anti-HA (red) or epifluorescence of the GFP (green) on the PfPRP2-RFA tagged line shows an identical punctate pattern. Scale bar represents 5μm. Nuclei of parasites were stain with DAPI (blue).

Analysis of the primary sequence of PfPRP2 (PlasmoDB: PF3D7_1320000, www.plasmodb.org) failed to identify any recognizable protein domains apart from a signal peptide allowing entry in to the secretory pathway (Fig 1A, amino acids 1–20). BLAST analysis (http://blast.ncbi.nlm.nih.gov/Blast.cgi) using the full length PfPRP2 sequence revealed that only other *Plasmodium* species had proteins highly homologous to the whole length of PfPRP2. Hypothetical proteins with homology to the C-terminus of PfPRP2 (from around amino acid 700) were found but only in a subset of apicomplexan parasites (*Cryptosporidium*, *Neospora*, *Hammondia*, *Toxoplasma*, *Eimeria* and also *Plasmodium* (S1 Table)). Furthermore orthoMCL analysis (www.orthomcl.org) showed no orthologues outside of plasmodia (not shown). Interestingly, for all *Plasmodium* species apart from *P*. *falciparum* and *P*. *reichenowi*, the homology drops for a short region in the middle of PfPRP2, in between the *Plasmodium* specific N-terminus and the more broadly conserved C-terminus. It is tempting to speculate that addition of the N-terminal module fulfills a function specific to plasmodia, perhaps associated with their requirement for erythrocytes as host cells.

### PfPRP2 localizes to the Golgi apparatus throughout the blood stages

To investigate the role of PfPRP2 in the erythrocytic cycle, we first tagged the endogenous locus with a regulatable fluorescent affinity tag (RFA) by single cross-over recombination [17](Fig 1B). The RFA tag is based on an *E*. *coli* DHFR degradation domain (DDD) fused to an HA epitope and GFP. In the absence of trimethoprim (TMP), a folate analogue, the fusion protein is destabilized and then targeted to the proteasome for degradation [18]. This system has been successfully used to demonstrate that the *P*. *falciparum* proteins Proteasome lid regulatory subunit 6 (PfRPN6) and PfHSP110c were essential for the erythrocytic stage[17, 19]. Clones expressing PfPRP2-RFA were obtained after 3 cycles on and off the selection drug BSD and used for subsequent analyses (Fig 1C and 1D). An anti-HA Western blot on total protein extracts of saponin-lysed parasites reveals a single band at around 150 kDa (Fig 1E). This is smaller than the expected size of 195 kDa for PfPRP2-RFA and suggests that the protein is potentially N-terminally processed. To assess the subcellular localization of the protein, an immunofluorescence assay (IFA) was performed using an anti-HA antibody on schizont stage parasites. A typical punctate pattern consistent with the proposed apical tip localization was observed for both the anti-HA antibody and the native GFP fluorescence, in line with PfPRP2 being a potential rhoptry protein (Fig 1F)[13]. The addition of a tag to a protein can sometimes result in mislocalization for a number of reasons such as misfolding for example. Indeed, misfolded tagged proteins sometimes remain trapped in the endoplasmic reticulum (ER) but the absence of consistent overlap between the ER tracker labeling around the DAPI stained nucleus and the PfPRP2-RFA dots demonstrates that this is not the case here (S1 Fig). In addition, because the initial work on *P*. *berghei* PRP2 showed that it is most likely an essential protein and that the parasite tolerated the addition of the GFP tag[13], we believe that the same is potentially true for *P*. *falciparum* i. e. that if the RFA tag were to have an effect on the PfPRP2 localization, this would likely not be tolerated because of the essentiality of the protein. This thus makes us more confident that PfPRP2-RFA is properly localized.

**Fig 2:**
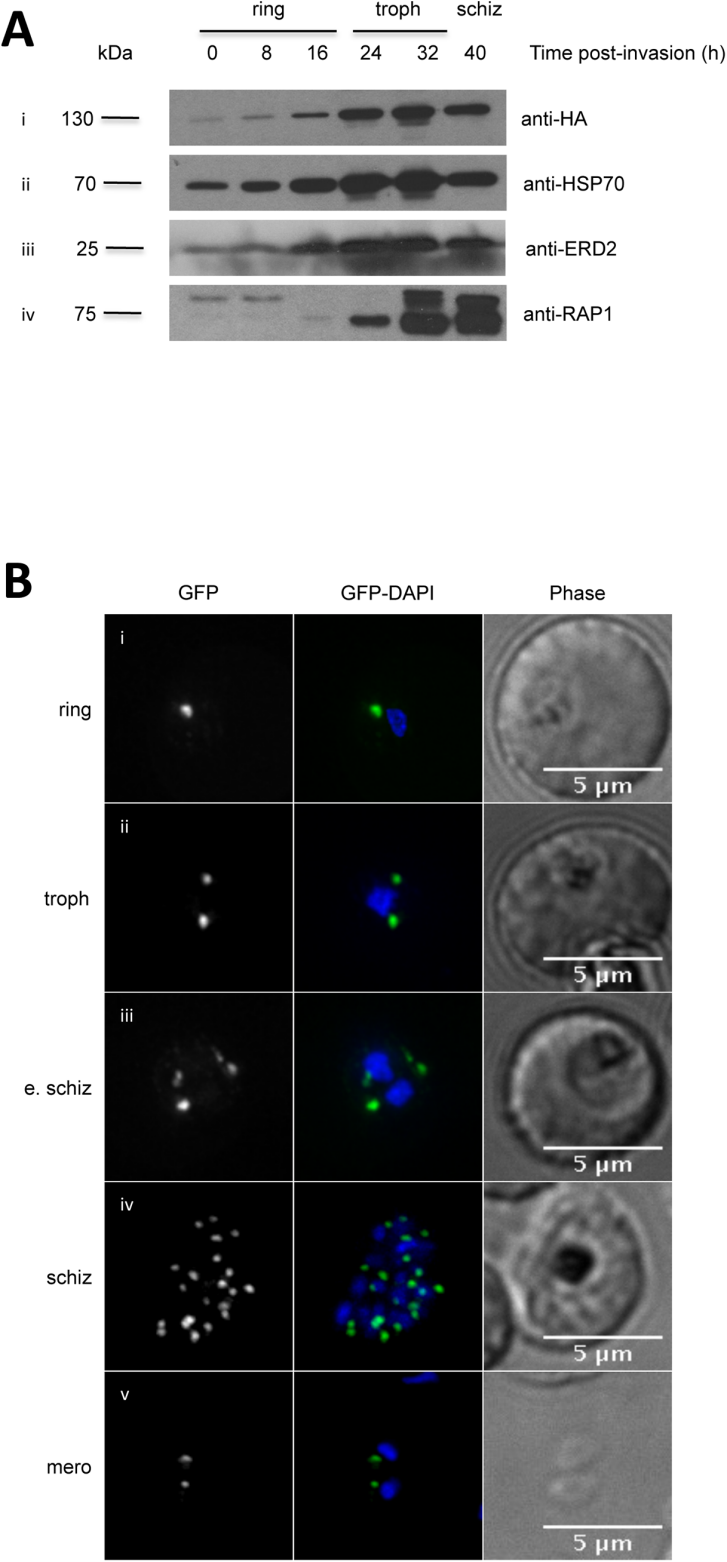
PfPRP2-RFA is constitutively expressed throughout the erythrocytic cycle. (A) Western blots on parasite extracts from tightly synchronous parasites show that PfPRP2-RFA is detected throughout the blood stages (Ai, anti-HA) similar to the constitutive cytosolic protein HSP70 (Aii) and the constitutive Golgi protein ERD2(Aiii). The rhoptry protein RAP1 is detected in very early and late stage parasites (Aiv). (B) Dynamics of the localization of PfPRP2-RFA throughout the asexual stages. In ring stage parasites, a single dot of fluorescence is seen close to the nucleus (Bi). In trophozoites, the dot starts to multiply, prior to nuclear division (Bii). Multiplication of the PfPRP2-RFA signal continues once nuclear division is undertaken (Biii, Biv). In free merozoites, each parasite inherits one locus of PfPRP2-RFA fluorescence (Bv). The fluorescence of PfPRP2-RFA is pseudocolored in green and the nuclei of parasites were stained with DAPI (blue). Scale bar represents 5μm. Troph: trophozoite; E. schiz: early schizont; Schiz: schizont; Mero: merozoite.

To investigate the expression profile of PfPRP2-RFA throughout the erythrocytic cycle, tightly synchronous parasites were collected every 8 hours from the ring to the schizont stage. Western blots on total protein extracts from an equal number of saponin-lysed parasites show that PfPRP2-RFA is expressed throughout the blood stages, with the strength of the signal increasing from ring to late trophozoite/schizont stages, similar to Heat shock protein 70 (HSP70) and ER lumen protein retaining receptor (ERD2), constitutively expressed proteins (Fig 2Ai, ii and iii)[13, 20–22]. These results correlate with the various studies of mass spectrometry-based evidence which have detected PfPRP2 peptides in rings[23], trophozoites[23–25] and schizonts[25, 26]. This pattern of protein expression is in contrast to most proteins known to be involved in the invasion process, like the Rhoptry associated protein 1 (RAP1), which are usually only expressed in the later schizont stage and carried over in young rings (Fig 2Aiv) [27]. Interestingly, the Rhoptry-associated membrane antigen (RAMA)[14], Pf34[15] and PfRhop148[16] are rhoptry proteins expressed from the trophozoite stage, before the appearance of the rhoptries, which led to the hypothesis that they might play a role in the biogenesis of these organelles. Intriguingly, when looking at the published RNA expression data of PfPRP2, one can see peaks of expression at the ring and schizont stages with a dip in the signal at the trophozoite stage[28, 29]. This non concordance between RNA and protein levels has previously been observed for other *P*. *falciparum* proteins such as the nuclear peroxiredoxin PfnPrx[30] and might potentially be explained, for example, by a low turnover rate of the protein. Of note, a recent study on the levels of steady-state mRNA and polysome-associated mRNA showed that though the levels of PfPRP2 mRNA were higher in ring stages, the polysome-associated levels remained constant throughout the blood stages suggesting that the proportion of actively translated PfPRP2 mRNA does not vary between rings, trophozoites and schizonts [31].

**Fig 3:**
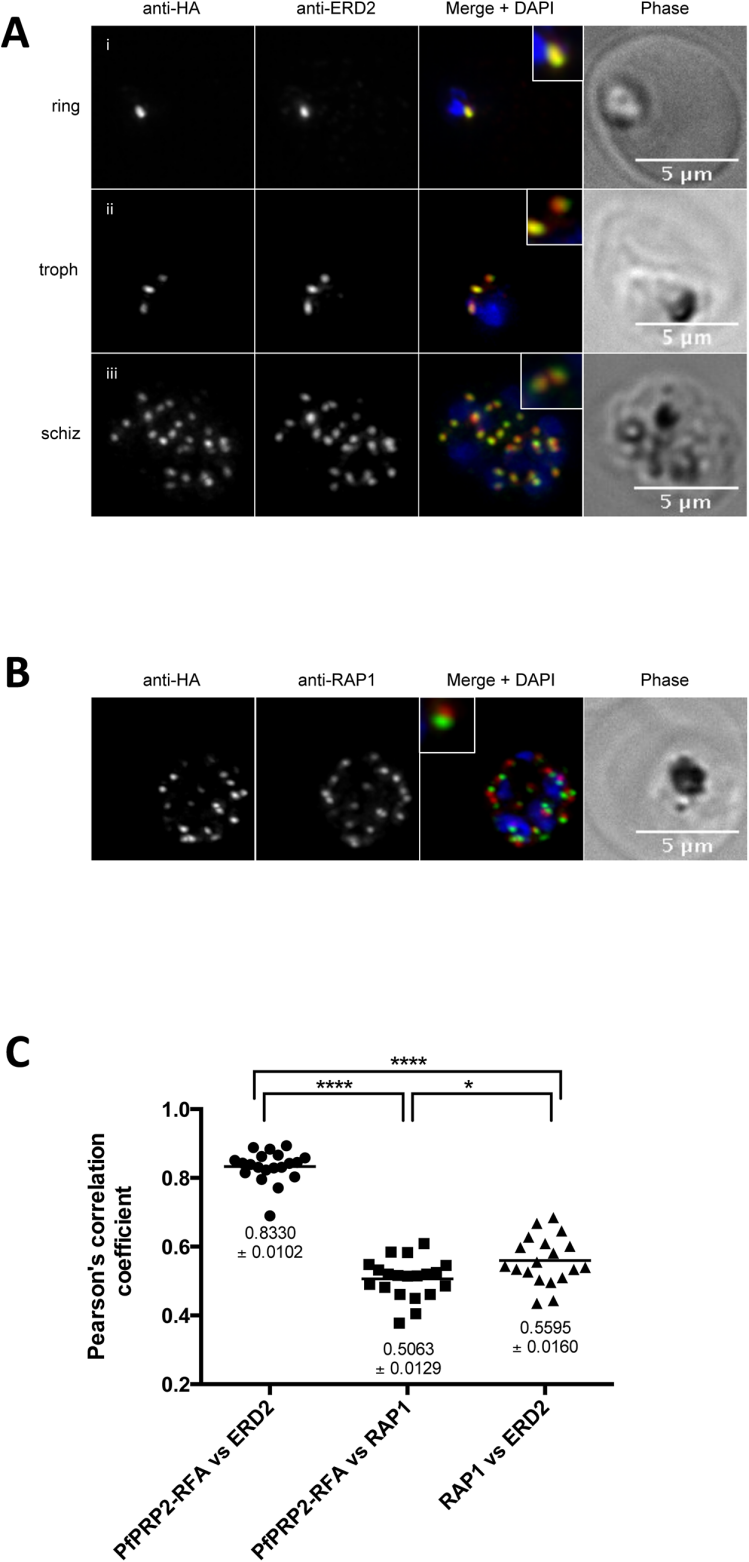
PfPRP2-RFA overlaps extensively with the Golgi apparatus throughout the erythrocytic cycle. (A) In ring stages, PfPRP2-RFA is found as a single punctate pattern colocalizing with the cis-Golgi marker ERD2 (Ai). In trophozoites, prior to nuclear division, the pattern associated with both PfPRP2-RFA and ERD2 becomes triple dots, showing the division of the Golgi, prior to nuclear replication (Aii). In schizont stages, the multiple PfPRP2-RFA signals still colocalize with the cis-Golgi protein ERD2 (Aiii). (B) In mature schizonts, PfPRP2-RFA is found in close proximity but does not overlap with the rhoptry marker RAP1. *RAP1*, rhoptry associated protein 1. Nuclei of parasites were stained with DAPI (blue). The fluorescence of PfPRP2-RFA is pseudocolored in green and other markers are in red. Scale bar represents 5μm. (C) Pearson’s correlation coefficients (r) of PfPRP2-RFA colocalization with the different organelle markers were calculated by the intensity correlation of Alexa fluor 488 and 594. Each dot represents an individual cell. Horizontal line represents the mean. The mean ± SEM are represented for each marker. PfPRP2-RFA vs RAP1, n = 20; PfPRP2-RFA vs ERD2, n = 20. ERD2 vs RAP1, n = 19. **** = *p*-value <0.0001; * = *p*-value = 0.013; *P*-values were calculated using a two-tailed Student’s t-test.

After ascertaining that PfPRP2-RFA was expressed throughout the erythrocytic cycle, we next looked at the dynamics of the subcellular distribution of PfPRP2-RFA by epifluorescence microscopy in live parasites. In early ring stages, one spot of fluorescence can be observed, close to the nucleus (Fig 2Bi). As the parasite matures to the trophozoite stage, before nuclear division has started, the single dot becomes 2 separate dots on either side of the nucleus (Fig 2Bii). This is in contrast to the *P*. *berghei* homologue of PRP2, to RAMA, and to PfRhop148 which are found as a diffuse and weak signal at this stage [13, 14, 16]. This diffuse staining was thought to represent the Golgi, in agreement with a previous report of a dispersed Golgi in *P*. *falciparum*[32] however this was challenged by subsequent work arguing for a more complex organellar architecture with a tight spatial association of markers of the transit ER (Sec13p), the cis-Golgi (Golgi re-assembly stacking protein (GRASP) and ERD2) and the trans-Golgi (Ras-related protein 6, RAB6) in ring and trophozoite stage parasites[33, 34]. Multiplication of the PfPRP2-RFA foci continues during nuclear division until the schizont stage is reached (Fig 2Biii and 2Biv)). In free merozoites, one spot of PfPRP2-RFA is observed in close proximity to nucleus (Fig 2Bv). This is reminiscent of the behavior of proteins associated with the Golgi apparatus like GRASP, ERD2 and RAB6 [32, 33, 35]. To further define the subcellular localization of PfPRP2-RFA, IFAs were performed using an anti-ERD2 antibody, a marker of the cis-Golgi [32]. Near perfect overlap between the fluorescence signals in rings, trophozoites and schizonts suggests that PfPRP2-RFA is localized to the Golgi apparatus during these stages (Fig 3A).

PbPRP2 was proposed to localize to the rhoptries based on its characteristic punctate pattern in schizonts however no colocalization with any apical markers were performed[13]. To determine whether the same was true in *P*. *falciparum*, we performed IFAs with an anti-RAP1 antibody [36], a marker of the rhoptry bulb. As shown in Fig 3B, though close apposition can be seen between PfPRP2-RFA and RAP1, no overlap was observed. To analyze this in a quantitative manner, Pearson's correlation analysis was performed on several cells. As shown in Fig 3C, the r coefficient of PfPRP2-RFA and ERD2 compared to PfPRP2-RFA and RAP1 is significantly higher (r coefficients of 0.8330±0.0102 vs 0.5063±0.0128, respectively). Despite the absence of extensive overlap between the fluorescence signals from their respective channel, the r value for PfPRP2-RFA vs RAP1 is still high. This can most likely be explained by the fact that because zero-zero pixels were included in our analysis and no threshold was applied, the r coefficients of separate, non-colocalizing organelles might still be substantial since most of the pixels coming from our images of schizont stage parasites are zero/background. This is supported by the fact that the r value of PfPRP2-RFA vs RAP1 is comparable with the r value of obtained for the Golgi (ERD2) and the rhoptries (RAP1), distinct, non-overlapping organelles (0.5063±0.0128 vs 0.5595±0.0160). Globally, our colocalization analyses suggest that PfPRP2-RFA localizes to the Golgi and not to the rhoptries. The proposed rhoptry localization of the *P*. *berghei* homologue of PRP2 was solely based on its punctate pattern in schizont stage parasites[13]. Perhaps performing the same type of colocalization analyses we did here with markers of the rhoptries and the Golgi, but this time in *P*. *berghei*, might potentially resolve this discrepancy and reveal the PbPRP2 is also not a rhoptry protein. The absence of clear apical staining in mature salivary gland sporozoites expressing PbPRP2-GFP is indeed further indication that, at least in sporozoites, the protein does not localize to the rhoptries[13].

To directly explore the role of PfPRP2 in the erythrocytic stages we attempted to conditionally knockdown its expression by removing the stabilizing ligand TMP from the culture medium. As seen in "Fig A in S2 Fig", no obvious decrease in the protein levels of PfPRP2-RFA signal, as detected using an anti-HA antibody, is seen after 24 hours without the drug. This is further demonstrated by comparing the densitometric ratio of PfPRP2-RFA with the control PfHSP70 ("Fig B in S2 Fig"). It is known that inhibition of parasite growth can sometimes be observed despite the absence of degradation of the RFA-tagged protein of interest and this led to the suggestion that destabilization of the protein could also prevent its correct function by proper formation and/or assembly of protein complexes [7, 19, 37]. To investigate whether this was the case for our PfPRP2-RFA line, growth curve analyses with and without TMP were performed. As shown in "Fig C in S2 Fig", no difference in parasitaemia is seen even after 72 hours off the TMP drug. Our failure to conditionally regulate the function of PfPRP2 with the DDD system might potentially be explained by the fact that because the protein possesses a signal peptide, it is likely co-translationally inserted into the ER and might therefore not be as accessible to proteosomal degradation as cytosolic proteins. This is not without precedent as a number *P*. *falciparum* and *T*. *gondii* proteins tagged with degradation systems failed to be knocked down [37–39]. That being said, conditional knockdown of FKBP-tagged PfRESA, a protein exported to the red blood cell cytosol, has been successfully achieved which shows that at least some non cytosolic proteins can be regulated with destabilization domains[38]. Perhaps other conditional expression systems not based on the destabilization of a protein of interest, such as the glmS riboswitch which functions at the RNA level, [40] and the inducible DiCre recombinase which cleaves any DNA fragment flanked by LoxP sites[41] will prove more useful in getting functional insight into the roles of proteins residing or transiting through the secretory pathway like PfPRP2. Indeed, the glmS ribozyme has been successfully used to decipher the function of key effectors of the *P*. *falciparum* protein export pathway such as the ER-resident Plasmepsin V[42] and the component of the parasitophorous vacuole membrane (PVM)-localized translocon PTEX150[6]. Our failure to knockdown the expression of PfPRP2 therefore does not allow us to speculate on its potential functions at this stage but we are currently attempting to use the glmS ribozyme system to hopefully answer this question. That being said, the inability of the *P*. *berghei* homologue PbPRP2 to be knocked out suggests that it is likely to be critical for the erythrocytic cycle.

### PfPRP2 is not secreted during the merozoite invasion process

**Fig 4:**
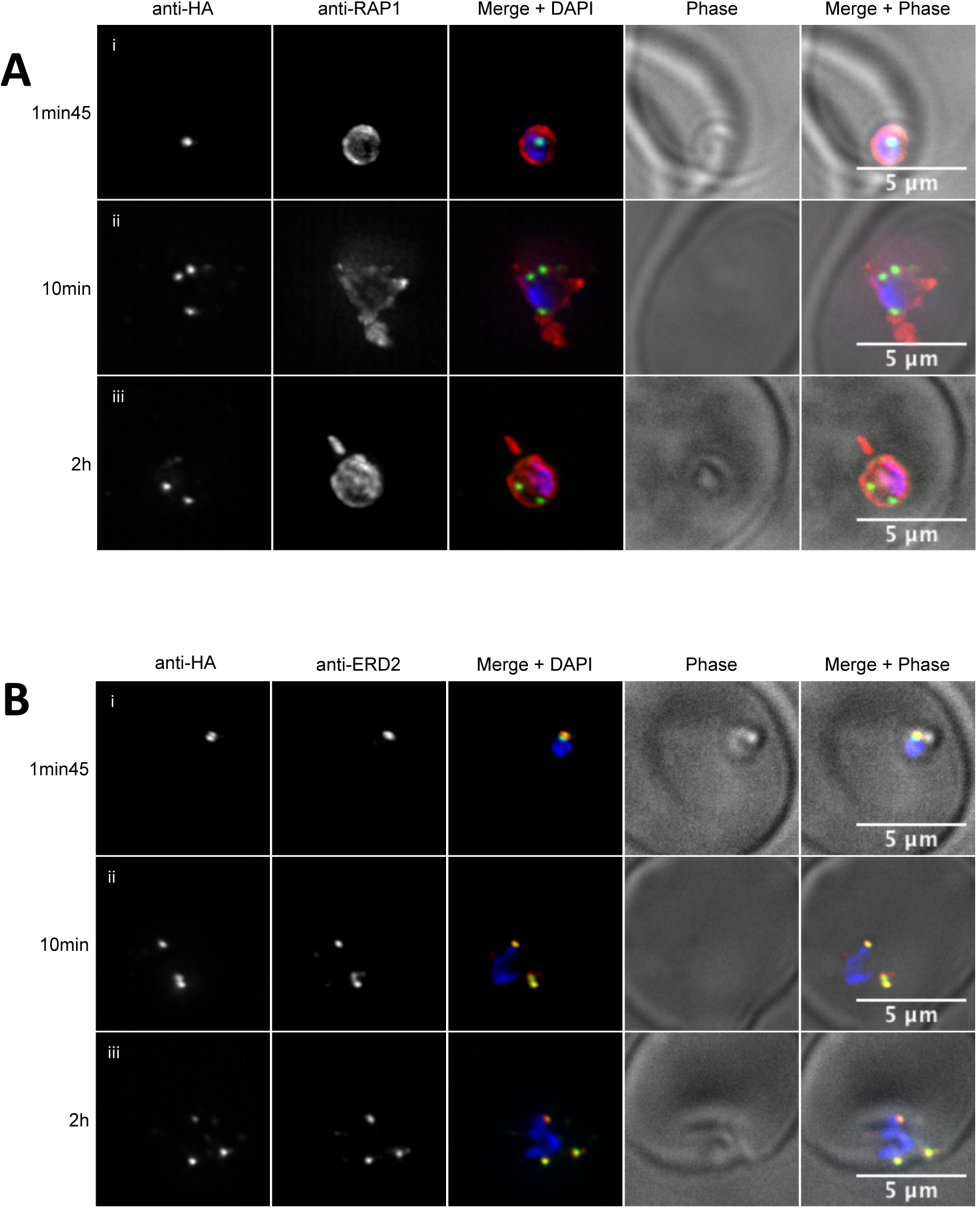
PfPRP2-RFA is not secreted during erythrocyte invasion. IFA time course analysis of the spatial distribution of PfPRP2-RFA during and after merozoite invasion. (A) Invading merozoites and post-invasion parasites are labelled with antibodies against the rhoptry bulb protein RAP1 (red) and PfPRP2-RFA using anti-HA (green). Shortly after invasion is completed, RAP1 is found secreted in the nascent PV tightly surrounding the parasite whilst PfPRP2-RFA remains in a discrete spot close to the nucleus (4Ai, 1min45). At later times following invasion (4Aii 10mins and iii, 2 hours post-invasion), RAP1 is now seen as pseudopodia-like and whorl-like extensions whilst PfPRP2-RFA shows a number of distinct spots, always inside the boundaries of the RAP1 signal. (B) Time course of invasion now labeled with antibodies against the cis-Golgi marker ERD2 (red) and PfPRP2-RFA using anti-HA (green). Invasion and post-invasion images show colocalization between PfPRP2-RFA and ERD2 with both proteins seen as a single dot close to the nucleus early after invasion and as subsequent distinct spots further on. Scale bar represents 5μm. Nuclei of parasites were stained with DAPI (blue).

Organelles of the apical complex are secreted at specific times during the invasion of an erythrocyte by *P*. *falciparum* merozoites and recent advances have allowed the visualization of some of the molecular players involved [43, 44]. To test whether PfPRP2-RFA is secreted during the invasion process as would be expected of a rhoptry protein, we performed IFAs on merozoites caught at different times of the process. To differentiate parasites that had completed invasion from parasites attached to the RBC membrane, we used RAP1 as a marker of rhoptry secretion/PV generation[44]. Rhoptries are compartmentalized and recent evidence suggests that the location of a protein within the rhoptry correlates with its order of secretion and its subsequent role during invasion with rhoptry tip proteins being involved in adhesion to the red blood cell surface, rhoptry neck proteins in the formation of the tight junction, rhoptry bulb proteins in the formation of the PV and PVM and finally rhoptry base and membrane proteins in a post PV/PVM role in development [8]. As seen in Fig 4Ai, early after invasion is completed, the rhoptry bulb containing RAP1 has been fully secreted in the PV surrounding the merozoite but PfPRP2-RFA can still be seen as a single dot close to the nucleus, showing that it had not been secreted with the contents of the rhoptry bulb and providing further evidence that PfPRP2-RFA is not a rhoptry bulb protein. Intriguingly, when looking at parasites at a longer time after invasion, from 10 minutes to 2 hours, the PfPRP2-RFA signal was often seen as several foci of fluorescence (Fig 4Aii to iii). Due to the extensive colocalization of PfPRP2-RFA with ERD2 throughout the erythrocytic stages, we decided to investigate whether that behavior could still be observed during the invasion process. The distribution of ERD2 was found to overlap extensively with that of PfPRP2-RFA at all stages of invasion (Fig 4B). Of note, several ERD2 foci are also observed when parasites are imaged more than 10 minutes after they are put in contact with the red blood cells. Whether these foci represent functional Golgis or a loss of the structural integrity of the organelle is unknown at this stage. We have tried to perform live cell imaging on recently invaded PfPRP2-RFA parasites to follow the fate of these foci and determine if the single fluorescence dot we observed in older rings is derived from these or newly generated organelles but have been unsuccessful so far. Nevertheless, our data demonstrate that, unlike other rhoptry proteins, PfPRP2-RFA is not secreted during the invasion process.

## Conclusion

In conclusion, the absence of colocalization of PfPRP2 with the rhoptry marker RAP1 coupled with the fact that it is not secreted during the invasion process demonstrate that it is not a rhoptry protein. Moreover, the extensive overlap with the Golgi marker ERD2 suggests that it could be part of this organelle. In light of this, we propose to tentatively change its name from Putative rhoptry protein 2 to Golgi protein 1. The fact that no PRP2 orthologues can be found outside of the *Plasmodium* genus might mean that its function is specifically required for the establishment of the parasite in its host cell, the erythrocyte. Our attempts at conditionally knocking down the expression of PfPRP2 using the DDD system having failed, this prevents us from speculating more on what this putative function might be however, the inability to genetically delete it's homologue in *P*. *berghei* suggests it is likely to be essential in the erythrocytic cycle.

## Materials and Methods

Study approved by the Canadian Blood Services (CBS) research ethics board, project number 2015.001 and by the CHU de Québec IRB, project number 2015–2230, B14-12-2230, SIRUL 104595. Written consent was obtained by the CBS for all study participants.

### Parasite Cultures

*P*. *falciparum* asexual stage parasites were maintained in human erythrocytes (blood group O+) at a hematocrit of 4% with 0.5%(w/v) Albumax^TM^(Invitrogen) [45]. *P*. *falciparum* 3D7 parasites were originally obtained from David Walliker at Edinburgh University. Cultures were synchronized by incubation with sorbitol as described previously [46]. For time course experiments, a mix stage culture of parasites was treated with sorbitol, put back in culture for 16 hours at which point it was synchronized a second time, resulting in late ring stage parasites spread over an 8 hour window (between 16 to 24 hours). The parasites were then put back in culture to mature and proceed through reinvasion at which point the harvesting for the time points was started and continued every 8 hours until the subsequent rupturing of schizonts.

To image merozoites during the process of invasion, viable filtered merozoites were prepared and used as described previously [43, 44, 47].

### Vector Construction, Transfection, and Southern Blotting

To create the plasmid used for the integration of the RFA at the 3'end of the *PfPrp2* gene by single crossover, a PCR fragment containing nucleotides 2819 to 3774 of *PfPrp2* without a stop codon was cloned into the Xho1-AvrII sites of the pGDB vector [17]. Because wild-type *P*. *falciparum* is sensitive to trimethoprim, the DHFRdd system requires the use of parasite strains resistant to this antifolate. The system was developed with a PfPlasmepsin 1 knock out strain (ΔPM1) (PfPM1 is a non essential gene) expressing hDHFR, rendering the parasites resistant to TMP[17] so we decided to use this same strain for our studies. Parasites were transfected, and integrants were selected as described previously[48]. Briefly, *P*. *falciparum* 3D7 ΔPM1 parasites [49] were transfected with 100 ug of purified plasmid DNA (Promega). Positive selection for transfectants was achieved using 2.5 mg/ml BSD (Sigma-Aldrich) and 5 uM TMP (Sigma-Aldrich). Integration was monitored by Southern blots according to standard procedures.

### Western blotting

For immunoblots, saponin-lysed parasite pellets from highly synchronous PfPRP2-RFA parasites were separated in sample buffer on 7% (w/v) SDS-PAGE gels under reducing conditions and transferred to PVDF membranes (Millipore). Antibodies (mouse monoclonal anti-Aldolase 1:200 (Immunology Consultants Laboratory, MB720); rabbit polyclonal anti-PfHSP70 1:20000 (StressMarq Biosciences Inc, SPC-186C); mouse monoclonal anti-RAP1 1:5000 [36]; mouse monoclonal anti-HA 1:2000 (Cedarlane, clone HA.C5); rabbit polyclonal anti-ERD2 1:2000 [22]) were diluted in 0.1% (v/v) Tween 20-phosphate-buffered saline with 4% (w/v) skim milk. Appropriate HRP-coupled secondary antibodies were used and immunoblots were revealed by ECL (Amersham Biosciences). For the time course of expression analysis, proteins extracted from an equal number of cells were used for each time point.

### Fluorescence Imaging

Fluorescence images of parasites were captured using an Applied Precision Deltavision Elite microscope with a sCMOS camera and analyzed with the SoftWorx software. Pearson's correlation coefficients were calculated with the Softworx software on regions of interests of image stacks, including zero-zero pixels and without thresholding. Chromatic calibration of the microscope was performed prior to imaging experiments. For immunofluorescence assays, parasites were fixed using 4% paraformaldehyde (ProSciTech) and 0.0075% glutaraldehyde (ProSciTech) as previously described [50]. After blocking in 3% bovine serum albumin (Sigma) the cells were incubated for 1 hour with rabbit anti-ERD2 1:2000 [22]; mouse monoclonal anti-RAP1 1:2000 [36]; rat monoclonal anti-HA 1:2000 (Roche, 3F10). Bound antibodies were then visualised with Alexa Fluor-594 anti-rabbit IgG or anti-mouse IgG (Molecular Probes) diluted 1:1000. Parasites were mounted in Vectashield (Vecta Laboratories) containing with 0.1 μg/ml 4', 6–diamidino-2-phenylindole (DAPI, Invitrogen). For live cell imaging of the ER, PfPRP2-RFA parasites were incubated with 1mM ER tracker (Molecular Probes E34250) for 30 min at 37°C and DAPI was added for the final 5 min.

### Conditional knockdown analysis

For conditional knockdown analysis, tightly synchronous ring stage PfPRP2-RFA parasites were incubated with or without 5uM TMP for 24 hours, until they reached the schizont stage, after which the parasites were harvested, saponin-lysed and the proteins extracted in SDS sample buffer and analyzed by Western blot. To investigate the effect of the absence of TMP on parasite growth, tightly synchronous ring stage PfPRP2-RFA parasites were seeded at 0.5% parasitaemia and grown on or off 5uM TMP. Total parasitaemia was calculated by fluorescence activated cell sorting (FACS) on a BD FACSCanto A. Briefly cells were harvested after 24, 48 and 72 hours in culture, stained with SYBRGold (Invitrogen-Molecular Probe), fixed with 1% paraformaldehyde for 1 hour at 4°C after which 100 000 events were acquired on the FACSCanto A using the FACSDiva software. The data was analyzed with the FlowJo software.

## Supporting Information

S1 Fig PfPRP2-RFA minimally overlaps with the endoplasmic reticulum during the erythrocytic stages. (DOCX)

S2 Fig Absence of TMP does not result in the degradation of PfPRP2-RFA and has no effect on parasite growth. (DOCX)

S1 Table Results of the BLASTP analysis using the full length PfPRP2 sequence. (DOCX)
