# Supplementary material for: Evidence that the Malaria Parasite Plasmodium falciparum Putative Rhoptry Protein 2 Localizes to the Golgi Apparatus throughout the Erythrocytic Cycle
Source: PLoS One. 2015 Sep 16;10(9):e0138626. doi: 10.1371/journal.pone.0138626 (PMC4574476; doi:10.1371/journal.pone.0138626)
Supplement: S1 Fig — (DOCX) [file pone.0138626.s001.docx]

**Supplementary Figure 1. PfPRP2-RFA minimally overlaps with the endoplasmic reticulum during the erythrocytic stages.**

Live PfPRP2-RFA parasites were incubated with ER Tracker (red) and DAPI (blue). PfPRP2-RFA is seen with the GFP fluorescence (green). A) In ring stages, the ER surrounds the DAPI stained nucleus without overlapping the PfPRP2-RFA signal. B) In trophozoites, the ER has now started to elongate and become more reticulated while the PfPRP2-RFA punctate structure has started to be replicated. Both PfPRP2-RFA labeled structures now potentially seem in close apposition with the ER. C) At the schizont stage, after cellular division, the nuclei of individual merozoites are surrounded by the ER and the PfPRP2-RFA is still seen as puncta of fluorescence. In some merozoites but not all, the PfPRP2-RFA signal seems in close apposition and sometimes even overlap with the ER.
