# Supplementary material for: Evidence that the Malaria Parasite Plasmodium falciparum Putative Rhoptry Protein 2 Localizes to the Golgi Apparatus throughout the Erythrocytic Cycle
Source: PLoS One. 2015 Sep 16;10(9):e0138626. doi: 10.1371/journal.pone.0138626 (PMC4574476; doi:10.1371/journal.pone.0138626)
Supplement: S2 Fig — (DOCX) [file pone.0138626.s002.docx]

**Supplementary Figure 2. Absence of TMP does not result in the degradation of PfPRP2-RFA and has no effect on parasite growth.**

A) Western blot on total protein extracts from ring stage PfPRP2-RFA parasites incubated with and without TMP for 24 hours, until they reached the end of the schizont stage, shows that the absence of TMP does not lead to the degradation of PfPRP2-RFA. An anti-HA antibody was use to detect PfPRP2-RFA. B) Ratio of PfPRP2-RFA on PfHSP70 calculated by densitometry analysis of the Western blot signals from A). C) Growth curve of PfPRP2-RFA parasite strain with and without TMP shows no significant decrease in parasitaemia in absence of the stabilizing ligand. Error bars represent SEM of 3 independent biological replicates.
