# Supplementary material for: Evidence that the Malaria Parasite Plasmodium falciparum Putative Rhoptry Protein 2 Localizes to the Golgi Apparatus throughout the Erythrocytic Cycle
Source: PLoS One. 2015 Sep 16;10(9):e0138626. doi: 10.1371/journal.pone.0138626 (PMC4574476; doi:10.1371/journal.pone.0138626)
Supplement: S1 Table — (DOCX) [file pone.0138626.s003.docx]

**Supplementary Table 1. Results of the BLASTP analysis using the full length PfPRP2 sequence.**

| Organism | Strain | Gene ID | Score | E-Value | Homology region |
| --- | --- | --- | --- | --- | --- |
| *Plasmodium falciparum* | *3D7* | [PF3D7_1320000](http://eupathdb.org/eupathdb/showRecord.do?name=GeneRecordClasses.GeneRecordClass&source_id=PF3D7_1320000&project_id=PlasmoDB) | 2548 | 0.00E+00 | FL |
|  | *IT* | [PFIT_1319200](http://eupathdb.org/eupathdb/showRecord.do?name=GeneRecordClasses.GeneRecordClass&source_id=PFIT_1319200&project_id=PlasmoDB) | 2543 | 0.00E+00 | FL |
| *Plasmodium reichenowi* | *CDC* | [PRCDC_1319000](http://eupathdb.org/eupathdb/showRecord.do?name=GeneRecordClasses.GeneRecordClass&source_id=PRCDC_1319000&project_id=PlasmoDB) | 2315 | 0.00E+00 | FL |
| *Plasmodium vivax* | *Sal-1* | [PVX_122810](http://eupathdb.org/eupathdb/showRecord.do?name=GeneRecordClasses.GeneRecordClass&source_id=PVX_122810&project_id=PlasmoDB) | 1600 | 0.00E+00 | FL |
| *Plasmodium berghei* | *ANKA* | [PBANKA_141830](http://eupathdb.org/eupathdb/showRecord.do?name=GeneRecordClasses.GeneRecordClass&source_id=PBANKA_141830&project_id=PlasmoDB) | 1417 | 0.00E+00 | FL |
| *Plasmodium yoelii* | *yoelii 17X* | [PY17X_1420000](http://eupathdb.org/eupathdb/showRecord.do?name=GeneRecordClasses.GeneRecordClass&source_id=PY17X_1420000&project_id=PlasmoDB) | 1415 | 0.00E+00 | FL |
|  | *yoelii YM* | [PYYM_1421800](http://eupathdb.org/eupathdb/showRecord.do?name=GeneRecordClasses.GeneRecordClass&source_id=PYYM_1421800&project_id=PlasmoDB) | 1415 | 0.00E+00 | FL |
| *Plasmodium chabaudi* | *chabaudi* | [PCHAS_142010](http://eupathdb.org/eupathdb/showRecord.do?name=GeneRecordClasses.GeneRecordClass&source_id=PCHAS_142010&project_id=PlasmoDB) | 1400 | 0.00E+00 | FL |
| *Plasmodium yoelii* | *yoelii 17XNL* | [PY03873](http://eupathdb.org/eupathdb/showRecord.do?name=GeneRecordClasses.GeneRecordClass&source_id=PY03873&project_id=PlasmoDB) | 1253 | 0.00E+00 | FL |
| *Plasmodium knowlesi* | *H* | [PKH_141900](http://eupathdb.org/eupathdb/showRecord.do?name=GeneRecordClasses.GeneRecordClass&source_id=PKH_141900&project_id=PlasmoDB) | 791 | 0.00E+00 | N |
| *Plasmodium cynomolgi* | *B* | [PCYB_142910](http://eupathdb.org/eupathdb/showRecord.do?name=GeneRecordClasses.GeneRecordClass&source_id=PCYB_142910&project_id=PlasmoDB) | 706 | 0.00E+00 | C |
|  |  | [PCYB_142900](http://eupathdb.org/eupathdb/showRecord.do?name=GeneRecordClasses.GeneRecordClass&source_id=PCYB_142900&project_id=PlasmoDB) | 254 | 3.00E-67 | FL |
| *Neospora caninum* | *Liverpool* | [NCLIV_005500](http://eupathdb.org/eupathdb/showRecord.do?name=GeneRecordClasses.GeneRecordClass&source_id=NCLIV_005500&project_id=ToxoDB) | 203 | 3.00E-51 | C |
| *Toxoplasma gondii* | *GT1* | [TGGT1_222100](http://eupathdb.org/eupathdb/showRecord.do?name=GeneRecordClasses.GeneRecordClass&source_id=TGGT1_222100&project_id=ToxoDB) | 199 | 5.00E-50 | C |
|  | *ME49* | [TGME49_222100](http://eupathdb.org/eupathdb/showRecord.do?name=GeneRecordClasses.GeneRecordClass&source_id=TGME49_222100&project_id=ToxoDB) | 199 | 4.00E-50 | C |
|  | *VEG* | [TGVEG_222100](http://eupathdb.org/eupathdb/showRecord.do?name=GeneRecordClasses.GeneRecordClass&source_id=TGVEG_222100&project_id=ToxoDB) | 199 | 8.00E-50 | C |
| *Hammondia hammondi* | *H.H.34* | [HHA_222100](http://eupathdb.org/eupathdb/showRecord.do?name=GeneRecordClasses.GeneRecordClass&source_id=HHA_222100&project_id=ToxoDB) | 198 | 1.00E-49 | C |
| *Cryptosporidium muris* | *RN66* | [CMU_031840](http://eupathdb.org/eupathdb/showRecord.do?name=GeneRecordClasses.GeneRecordClass&source_id=CMU_031840&project_id=CryptoDB) | 186 | 7.00E-46 | C |
| *Cryptosporidium parvum* | *Lowa II* | [cgd5_170](http://eupathdb.org/eupathdb/showRecord.do?name=GeneRecordClasses.GeneRecordClass&source_id=cgd5_170&project_id=CryptoDB) | 181 | 4.00E-44 | C |
| *Plasmodium reichenowi* | *CDC* | [PRCDC_1318900](http://eupathdb.org/eupathdb/showRecord.do?name=GeneRecordClasses.GeneRecordClass&source_id=PRCDC_1318900&project_id=PlasmoDB) | 168 | 4.00E-40 | C |
| *Eimeria falciformis* | *Bayer Haberkorn 1970* | [EfaB_PLUS_5792.g561](http://eupathdb.org/eupathdb/showRecord.do?name=GeneRecordClasses.GeneRecordClass&source_id=EfaB_PLUS_5792.g561&project_id=ToxoDB) | 166 | 2.00E-39 | C |
| *Plasmodium falciparum* | *IT* | [PFIT_1319100](http://eupathdb.org/eupathdb/showRecord.do?name=GeneRecordClasses.GeneRecordClass&source_id=PFIT_1319100&project_id=PlasmoDB) | 163 | 1.00E-38 | C |
| *Plasmodium falciparum* | *3D7* | [PF3D7_1319900](http://eupathdb.org/eupathdb/showRecord.do?name=GeneRecordClasses.GeneRecordClass&source_id=PF3D7_1319900&project_id=PlasmoDB) | 161 | 4.00E-38 | C |
| *Plasmodium vivax* | *Sal-1* | [PVX_122805](http://eupathdb.org/eupathdb/showRecord.do?name=GeneRecordClasses.GeneRecordClass&source_id=PVX_122805&project_id=PlasmoDB) | 157 | 7.00E-37 | C |
| *Cryptosporidium muris* | *RN66* | [CMU_013110](http://eupathdb.org/eupathdb/showRecord.do?name=GeneRecordClasses.GeneRecordClass&source_id=CMU_013110&project_id=CryptoDB) | 152 | 2.00E-35 | C |
| *Eimeria brunetti* | *Houghton* | [EBH_0041040](http://eupathdb.org/eupathdb/showRecord.do?name=GeneRecordClasses.GeneRecordClass&source_id=EBH_0041040&project_id=ToxoDB) | 137 | 2.00E-30 | C |
| *Eimeria acervuline* | *Houghton* | [EAH_00021140](http://eupathdb.org/eupathdb/showRecord.do?name=GeneRecordClasses.GeneRecordClass&source_id=EAH_00021140&project_id=ToxoDB) | 136 | 3.00E-30 | C |
| *Eimeria necatrix* | *Houghton* | [ENH_00023930](http://eupathdb.org/eupathdb/showRecord.do?name=GeneRecordClasses.GeneRecordClass&source_id=ENH_00023930&project_id=ToxoDB) | 109 | 5.00E-22 | C |
| *Eimeria tenella* | *Houghton* | [ETH_00003025](http://eupathdb.org/eupathdb/showRecord.do?name=GeneRecordClasses.GeneRecordClass&source_id=ETH_00003025&project_id=ToxoDB) | 103 | 4.00E-21 | C |

BLASTP analysis performed on the NCBI website: http://blast.ncbi.nlm.nih.gov/Blast.cgi Gene ID: From www.plasmodb.org. FL: Full length; N: N-terminus; C: C-terminus.
